# Relationships Among Adipose Tissue Distribution, Vitamin D, and Bone Metabolism in Normoglycemic and Type 2 Diabetic Individuals

**DOI:** 10.3390/metabo16060379

**Published:** 2026-05-31

**Authors:** Tian-Hang Ma, Juan Zhao, Kun-Hou Zhou, Ya-Xin Guan, Fan Zuo, Xin Nian, Yi Zheng, Wen-Jiao Wang, Li-Juan Zhang, Tsutomu Kazumi, Jingshan Huang, Bin Wu

**Affiliations:** 1 First Affiliated Hospital of Kunming Medical University, 295 Xichang Road, Kunming 650032, China; 20240486@kmmu.edu.cn (T.-H.M.); zhaojuantuzi@163.com (J.Z.); guanyaxin@kmmu.edu.cn (Y.-X.G.); yiyanjing816@163.com (F.Z.); nianxin@kmmu.edu.cn (X.N.); 20220572@kmmu.edu.cn (Y.Z.); 20230615@kmmu.edu.cn (W.-J.W.); 20230616@kmmu.edu.cn (L.-J.Z.); 2 Xi’an Jiaotong-Liverpool University, Suzhou 215123, China; kunhou.zhou22@student.xjtlu.edu.cn; 3 Research Institute for Nutrition Sciences, Mukogawa Women’s University, Ikebiraki-Cho, Nishinomiya 663-8558, Hyogo, Japan; kazumi@mukogawa-u.ac.jp; 4 School of Computing, College of Medicine, University of South Alabama, Mobile, AL 36688, USA; huang@southalabama.edu

**Keywords:** SAT, VAT, 25(OH)D, 1,25(OH)_2_D_3_, BMD

## Abstract

Objectives: To investigate the interplay between adipose distribution, vitamin D metabolites, and bone mineral density (BMD) in Normal Glucose Tolerance (NGT) and type 2 Diabetic (T2DM) individuals. Methods: 167 participants (NGT: 61; T2DM: 106) were enrolled. Serum 25(OH)D, 1,25(OH)_2_D_3_, Parathyroid Hormone (PTH), and Ca were quantified. Visceral (VAT) and subcutaneous (SAT) adipose areas were assessed via dual bioelectrical impedance analysis. BMD and body composition were assessed via DXA. Metabolic indices (HOMA-IR, HOMA-β, ISI) were calculated. Results: 1. NGT: 25(OH)D was unrelated to adiposity. Conversely, 1,25(OH)_2_D_3_ was correlated inversely with VAT, SAT, body mass index (BMI), and Fat Mass Index (FMI), with VAT being the strongest independent predictor. 2. T2DM: High VAT correlated with insulin resistance yet paradoxically higher BMD. 25(OH)D correlated positively with Z-score, while 1,25(OH)_2_D_3_ correlated negatively with lumbar BMD. 3. VAT exerted a greater influence on insulin resistance than SAT, particularly in T2DM. Conclusions: 1. Visceral adiposity is the primary determinant of active 1,25(OH)_2_D_3_ metabolism in both NGT and T2DM individuals. 2. 1,25(OH)_2_D_3_ levels may be more closely associated with adiposity-related metabolic alterations than 25(OH)D. Despite lower 1,25(OH)_2_D_3_, the positive association between VAT and BMD in T2DM suggests complex mechanisms where visceral fat may paradoxically influence bone metabolism while driving insulin resistance.

## 1. Introduction

Vitamin D plays an essential physiological role in the human body, with adipose tissue serving as its primary reservoir. In the liver, vitamin D undergoes hydroxylation to form 25-hydroxyvitamin D 25(OH)D, which is subsequently converted in the kidneys by 1α-hydroxylase into 1,25-dihydroxyvitamin D [1,25(OH)_2_D_3_]. Among its metabolites, 1,25(OH)_2_D_3_ is the primary active form, exerting pleiotropic biological effects upon binding to the vitamin D receptor [1]. Clinical evidence suggests that vitamin D status is significantly influenced by metabolic conditions; for instance, a high Body Mass Index (BMI) is typically associated with lower circulating vitamin D due to its sequestration in expanded adipose depots [2]. Furthermore, type 2 diabetes mellitus (T2DM) and chronic hyperglycemia further exacerbate this deficiency by impairing the enzymatic activity of 1α-hydroxylase and altering vitamin D receptor expression. In the context of chronic diabetes, prolonged metabolic derangements can lead to a state of functional vitamin D insufficiency, which in turn contributes to systemic inflammation and exacerbated insulin resistance [3]. Beyond its well-established role in bone metabolism, vitamin D significantly influences adipocyte physiology. It modulates intracellular Ca^2+^ levels and the activity of Ca^2+^-dependent apoptotic proteases, thereby regulating adipogenesis and lipolysis while inducing apoptosis in mature adipocytes [4]. Furthermore, vitamin D can reduce intracellular lipid content and downregulate the expression and activity of adipogenic genes, ultimately enhancing adipocyte metabolic function [5]. These regulatory mechanisms have profound clinical implications, as the interplay between vitamin D and adipocyte dysfunction is closely linked to the development of metabolic syndrome and diabetic complications [6]. Although the link between vitamin D and obesity has been extensively studied, research exploring the specific relationship between 1,25(OH)_2_D_3_ adipose tissue distribution and bone metabolism remains sparse. In the present study, we quantified serum 25(OH)D and 1,25(OH)_2_D_3_ concentrations, lumbar bone mineral density (BMD), and visceral/subcutaneous adipose tissue areas across individuals with varying glycemic status to elucidate the correlations among these parameters.

## 2. Materials and Methods

### 2.1. Participants and Study Design

A total of 167 inpatients were recruited from the Department of Endocrinology at the First Affiliated Hospital of Kunming Medical University between May and August 2016. According to the 1999 World Health Organization (WHO) diagnostic criteria, participants were stratified into two groups: the Normal Glucose Tolerance (NGT) group (*n* = 61; fasting plasma glucose [FPG] < 6.1 mmol/L and 2h postprandial glucose [2h-PG] < 7.8 mmol/L) and the T2DM group (*n* = 106; typical diabetic symptoms plus random blood glucose ≥ 11.1 mmol/L, FPG ≥ 7.0 mmol/L, or 2h-PG after a 75 g glucose load ≥ 11.1 mmol/L). Eligible participants were unrelated Han Chinese individuals aged 20–70 years who had resided in the Yunnan region for over 10 years and maintained a stable body weight in the months preceding the study. The exclusion criteria included: (1) severe cardiac, hepatic, renal (e.g., Heart Failure, Acute Coronary Syndrome/Myocardial Infarction, Heart Failure, Uncontrolled Hypertension, Recent Cardiac Interventions, Severe hepatic/renal impairment), or parathyroid diseases; (2) acute diabetic complications (e.g., ketoacidosis, hyperosmolar hyperglycemic state) or severe chronic complications (e.g., diabetic foot, kidney failure); (3) severe osteoporosis, history of pathological fractures, or current treatment with calcitriol or bisphosphonates; (4) presence of cardiac pacemakers; (5) significant localized skin lesions; and (6) pregnancy.

### 2.2. Research Methods

#### 2.2.1. Clinical and Anthropometric Assessment

Comprehensive medical histories, including age, sex, and ethnicity, were documented for all participants upon enrollment. Standardized anthropometric measurements were performed to determine height, weight, waist circumference (WC), hip circumference (HC), and blood pressure.

#### 2.2.2. Assessment of Adipose Tissue Distribution

All participants underwent measurement of visceral fat area using the Omron DUALSCAN HDS-2000 (Kyoto, Japan) after an overnight fast of at least 8 h. With the participant in a supine position, abdominal cross-sectional area was measured at the umbilical level during breath-holding at expiration. Without changing position, dedicated trunk and limb electrodes were applied, and bioelectrical impedance analysis was performed during another breath-hold maneuver to automatically calculate the visceral fat area. Total body fat content was assessed via dual-energy X-ray absorptiometry (DXA) using the PRODIGY ADVANCE system (GE Healthcare, Chicago, IL, USA). Participants were further categorized into “excess-Excess VAT” (≥100 cm^2^) and “normal-Normal VAT” (<100 cm^2^) groups based on established clinical thresholds.

#### 2.2.3. Laboratory Assays

Fasting venous blood samples were collected from the antecubital vein. Serum calcium (Ca), FPG, alanine aminotransferase (ALT), aspartate aminotransferase (AST), blood urea nitrogen (BUN), serum creatinine (Scr), serum uric acid (SUA), total cholesterol (TC), triglycerides (TG), and high-density lipoprotein cholesterol (HDL-C) were measured using the OLYMPUS AU5400 automated biochemical analyzer (Olympus Corporation, Tokyo, Japan).

Parathyroid hormone (PTH), calcitonin (CT), 25(OH)D, glycated hemoglobin (HbA1c), insulin, and C-peptide were quantified using the MAGLUMI 400 automated chemiluminescence immunoassay system (SNIBE, Shenzhen, China). Serum levels of the active vitamin D metabolite, 1,25(OH)_2_D_3_, were determined using a high-sensitivity ELISA kit (Cusabio, Wuhan, China). (Detailed technical information regarding the 1,25(OH)_2_D_3_, 25(OH)D, PTH, HbA1C, C-peptide, and insulin Insulin assay is provided in the Supplementary Table S1).

#### 2.2.4. BMD and Fat Mass Index

Lumbar spine BMD and total body fat percentage (Fat%) were assessed using the DXA system (Prodigy Advance, GE Healthcare). For postmenopausal women or men aged ≥50 years, bone health was defined as: normal Normal (T-score > −1.0), osteopenia Osteopenia (−1.0 ≥ T-score ≥ −2.5), and osteoporosis Osteoporosis (T-score < −2.5). For premenopausal women and in men younger than 50 years, a Z-score ≤ −2.0 indicates that bone mass lower than expected for age.BMD values were recorded in g/cm^2^.

#### 2.2.5. Metabolic Indices and Calculations

Insulin dynamics and body composition indices were calculated using the following formulas:

HOMA-IR (Homeostasis Model Assessment of Insulin Resistance) = (FINS × FPG)/22.5 s

ISI (Insulin Sensitivity Index) = ln[22.5/(FPG × FINS)]

HOMA-β (Pancreatic β-cell Function Index) = 20 × FINS/(FPG − 3.5)

FMI (Fat Mass Index) = [Total Body Fat (%) × Body Weight (kg)]/Height^2^ (m^2^) × 100

### 2.3. Statistical Analysis

Statistical analyses were performed using SPSS version 26.0 and R version 4.6.0. Sample size was estimated a priori using G*Power software (3.1.9.6). For the primary analysis of the correlation between 1,25(OH)_2_D_3_ and visceral VAT, the sample size calculation was based on a two-sided alpha level of 0.05 and 80% power to detect a small-to-moderate to moderate correlation. Accordingly, a conservative target sample size of approximately 150 participants was considered sufficient [7]. A total of 167 participants were included in the final analysis, indicating adequate statistical power for the primary correlation analysis.

Continuous variables are presented as mean ± standard deviation or median (interquartile range), according to their distribution, and categorical variables are presented as numbers and percentages [8]. Pearson or Spearman correlation coefficients were used to assess the initial associations between parameters, as appropriate according to data distribution [9]. Partial correlation analyses were further performed to adjust for potential confounding factors, including age, sex, and BMI, and to estimate the independent associations among adipose tissue distribution, vitamin D metabolites, and BMD. The adjusted covariates were selected based on biological plausibility, prior evidence, and reporting recommendations for observational studies [10,11,12]. Diabetes status was additionally considered where appropriate, and diabetes duration was considered in analyses restricted to participants with type 2 diabetes mellitus. All tests were two-sided, and *p* values < 0.05 were considered statistically significant [8].

### 2.4. Ethical Approval and Consent to Participate

The protocol for this study was approved by the Ethics Committee of the First Affiliated Hospital of Kunming Medical University. All procedures performed in studies involving human participants were in accordance with the ethical standards of the institutional and/or national research committee and with the 1964 Helsinki Declaration and its later amendments.

## 3. Results

### 3.1. Comparison of Baseline Clinical Characteristics Between the NGT and T2DM Groups

A comparison of baseline characteristics was conducted between the NGT and T2DM group participants (Table 1). Statistically significant differences (*p* < 0.05) between the two groups based on their glycemic status were observed in age, height, the ratio of VAT to SAT area, and sex distribution.

### 3.2. Correlation Analysis of Serum 25(OH)D and 1,25(OH)_2_D_3_ with Clinical and Metabolic Parameters

In the NGT group, Spearman correlation analysis revealed that serum 25(OH)D levels were significantly and inversely associated with T3 and PTH. Notably, 1,25(OH)_2_D_3_ exhibited robust inverse correlations with nearly all primary adiposity metrics, including body weight, BMI, Body Fat (FAT%), Fat Mass Index (FMI), and both SAT and VAT areas.

Within the T2DM group, serum 25(OH)D demonstrated significant positive correlations with several bone quality markers, specifically Z3, Z4, Z (L1–L4), and Z (L2–L4). Conversely, it was negatively associated with VAT area and body weight.

**Figure 1:**
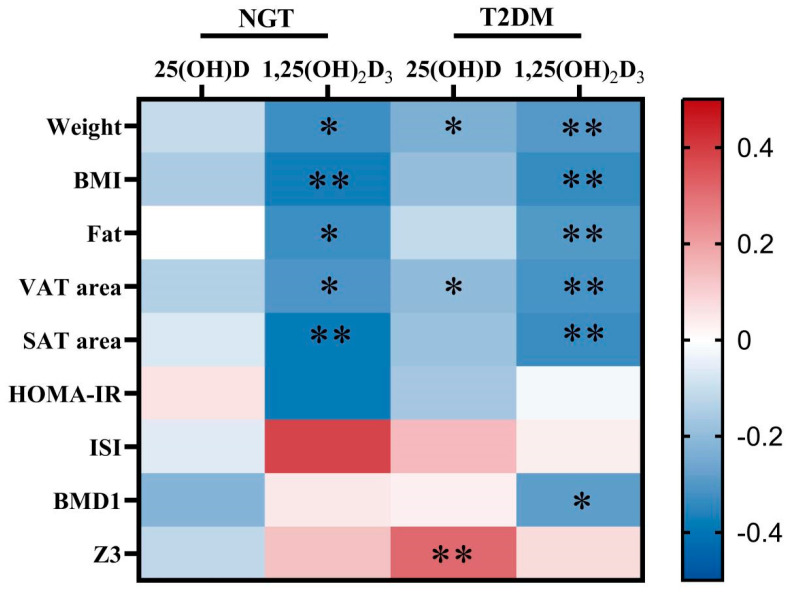
Correlation heatmap illustrating the associations between serum vitamin D metabolites and clinical/metabolic parameters in the NGT and T2DM groups. (Red indicates positive correlation; blue indicates negative correlation). *: *p* < 0.05, **: *p* < 0.01.

Furthermore, serum 1,25(OH)_2_D_3_ was extensively and negatively correlated with both adiposity and bone metabolism parameters. Specifically, inverse associations were observed with weight, BMI, Fat%, FMI, VAT area, SAT area, WC, HC, and WHR, as well as BMD indices including BMD1, BMD2, BMD3, BMD (L2–L4), and T-scores (L1, L2) (*p* < 0.05 for all; Table 2, Figure 1). Furthermore, sex-stratified analyses were conducted, yet no significant association between 1,25(OH)_2_D_3_ and BMD was observed in either the male or female subgroups (detailed data are presented in Supplementary Figure S1).

### 3.3. Correlation of VAT and SAT Areas with Clinical and Metabolic Parameters in the T2DM Group

Spearman correlation analysis within the T2DM group demonstrated that VAT area was significantly and positively associated with several anthropometric and metabolic markers, including height, weight, BMI, Fat%, FMI, the VAT/SAT ratio, WC, HC, WHR, and HOMA-IR. Conversely, VAT area exhibited a significant inverse correlation with the ISI. Regarding SAT area, positive associations were observed with height, weight, BMI, Fat%, FMI, WC, HC, and WHR, alongside a significant inverse relationship with the VAT/SAT ratio (*p* < 0.05 for all; Table 3).

Notably, in contrast to VAT, SAT area lacked any significant correlation with ISI (*p* > 0.05; Table 3).

### 3.4. Subgroup Analyses Based on VAT Area and BMI Thresholds

To further elucidate the metabolic impact of adipose distribution, participants in both the NGT and T2DM groups were stratified based on established clinical cut-offs. Subgroups were defined by VAT area (normal Normal VAT area: <100 cm^2^ vs. excess Excess VAT area: ≥100 cm^2^) and BMI (normal Normal BMI: <24 kg/m^2^ vs. excess Excess BMI: ≥24 kg/m^2^). Comprehensive comparisons of clinical and metabolic data between these groups were performed as follows.

#### 3.4.1. Comparison of Baseline Clinical Characteristics Stratified by VAT Area

Within the NGT group, a comparison of baseline clinical data revealed statistically significant differences in weight, BMI, Fat%, FMI, VAT area, SAT area, the VAT/SAT ratio, WC, HC, and WHR between the normal and excessive VAT groups. Similarly, in the T2DM group, subjects with excessive VAT differed significantly from their normal VAT counterparts across multiple clinical and adiposity parameters, including height, weight, BMI, Fat%, FMI, VAT area, SAT area, the VAT/SAT ratio, WC, HC, and WHR (all *p* < 0.05; Table 4).

#### 3.4.2. Comparison of Participant Characteristics Based on BMI Stratification

Across both NGT and T2DM groups, the overweight/obese BMI subgroup consistently demonstrated significantly higher adiposity indices (Weight, BMI, Fat%, FMI, VAT and SAT area, WC, HC, WHR) compared to their normal BMI counterparts (all *p* < 0.05; Table 5).

#### 3.4.3. Impact of Visceral Adiposity on Vitamin D and Bone Metabolism

**Figure 2:**
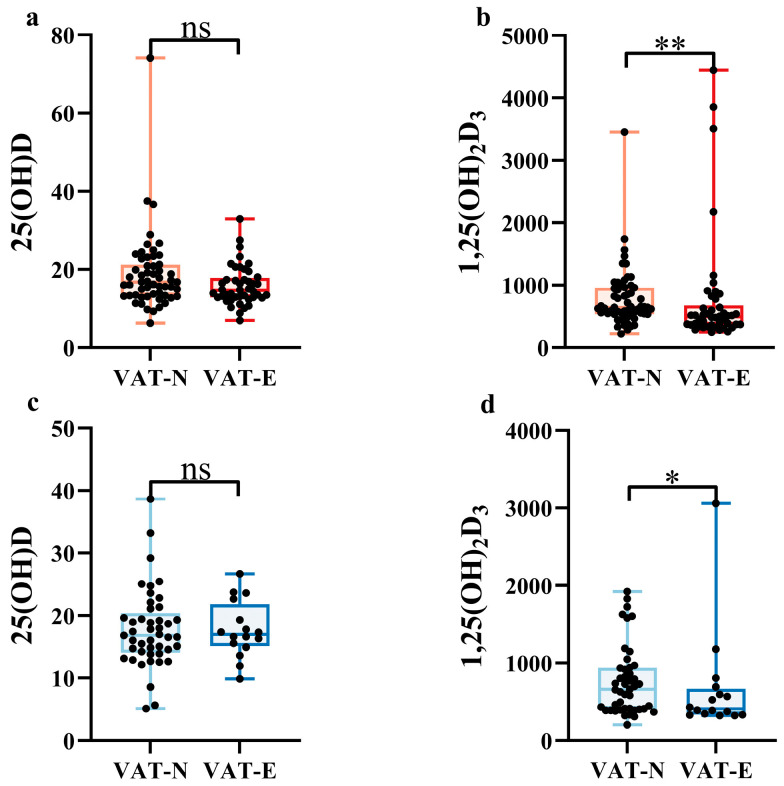
Comparison of serum 25(OH)D and 1,25(OH)_2_D_3_ concentrations in individuals stratified by VAT (VAT-N: Normal VAT Area; VAT-E: Excess VAT Area) area within the T2DM (**a**,**b**) and NGT (**c**,**d**) groups. ns: *p* > 0.05, *: *p* < 0.05, **: *p* < 0.01.

**Figure 3:**
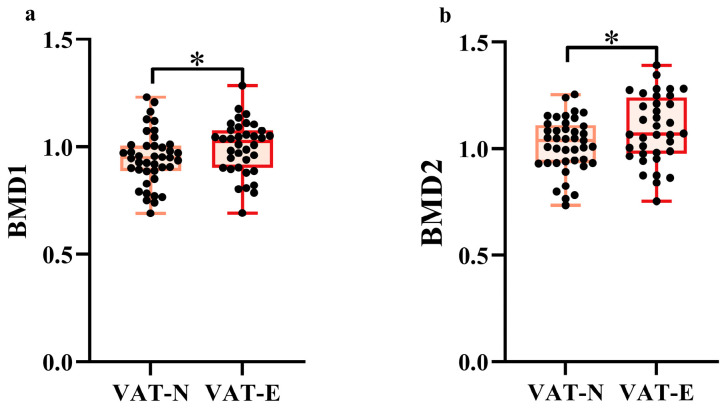
Comparison of lumbar spine BMD (BMD1 (**a**) and BMD2 (**b**)) across different VAT area within the T2DM group. *: *p* < 0.05.

In the NGT group, subjects with excessive VAT had significantly attenuated 1,25(OH)_2_D_3_ levels compared to the normal VAT group. In the T2DM group, the excess VAT area subgroup presented with exacerbated insulin resistance (higher HOMA-IR, lower ISI) and paradoxically higher BMD (BMD1, BMD2), alongside lower 1,25(OH)_2_D_3_ levels (all *p* < 0.05; Table 6, Figure 2 and Figure 3).

#### 3.4.4. Vitamin D and Bone Metabolism Profile by BMI Subgroups

**Figure 4:**
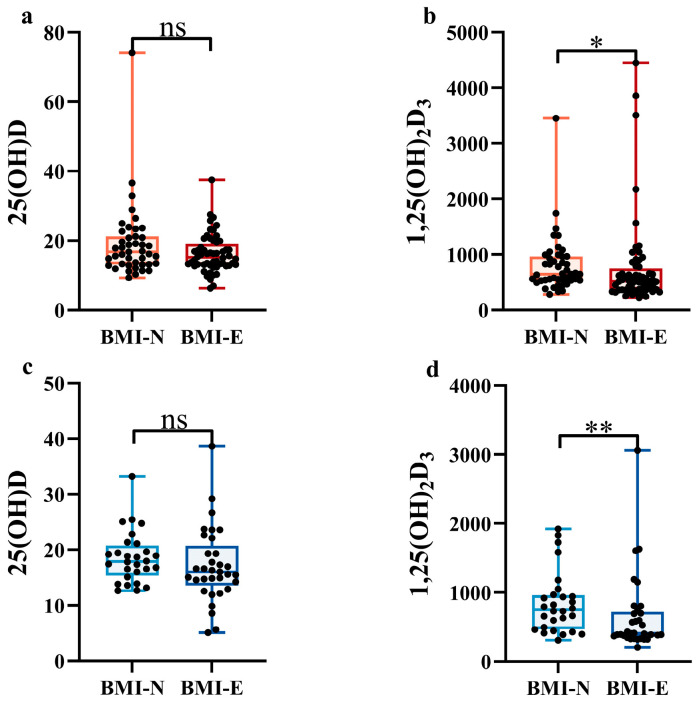
Comparison of serum 25(OH)D and 1,25(OH)_2_D_3_ concentrations among participants stratified by BMI levels (BMI-N: Normal BMI; BMI-E: Excess BMI) in the T2DM (**a**,**b**) and NGT (**c**,**d**) groups. ns: *p* > 0.05, *: *p* < 0.05, **: *p* < 0.01.

In the NGT group, a comparison of vitamin D, bone metabolism, and other clinical parameters between the normal BMI and overweight/obese subgroups revealed statistically significant differences in HOMA-IR, HOMA-ISI, BMD2, T2, and 1,25(OH)_2_D_3_. Similarly, within the T2DM group, the overweight/obese subgro-up exhibited significant differences in HOMA-IR, ISI, BMD1, BMD2, BMD (L1–L4), BMD (L2–L4), T2, and 1,25(OH)_2_D_3_ when compared to their normal BMI counterp-arts (all *p* < 0.05; Table 7, Figure 4).

### 3.5. Multivariable Linear Regression Analysis of Factors Associated with 1,25(OH)_2_D_3_

In the NGT group, correlation analysis revealed that 1,25(OH)_2_D_3_ was significantly and inversely correlated with weight, BMI, Fat%, FMI, VAT area, and SAT area. When these associated variables were entered into a multivariate regression analysis, only VAT area was retained in the final model as a significant independent predictor:

R^2^ = 0.092, F = 5.738

The resulting regression equation was formulated as:

1,25(OH)_2_D_3_ concentration = 957.389 − 0.333 × (VAT area)

**Figure 5:**
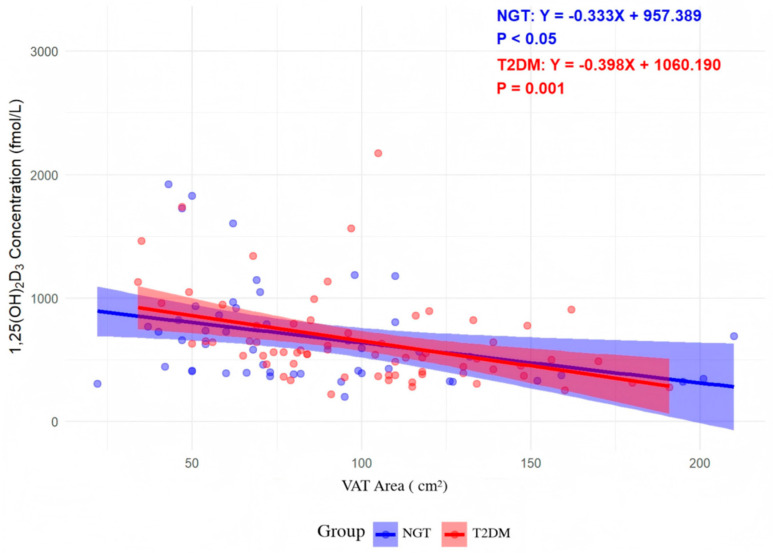
Scatter plots and linear regression lines showing the association between serum 1,25(OH)_2_D_3_ and VAT area across NGT and T2DM groups.

Similarly, within the T2DM group, 1,25(OH)_2_D_3_ exhibited significant associations with weight, BMI, Fat%, FMI, VAT area, SAT area, WC, HC, and WHR. Following a multivariate regression analysis incorporating these covariates, VAT area again emerged as the sole independent determinant remaining in the model (Table 8, Figure 5):

R^2^ = 0.145, F = 12.393

The regression model was defined as:

1,25(OH)_2_D_3_ concentration = 1060.190 − 0.398 × (VAT area)

## 4. Discussion

### 4.1. Relationship Between Adipose Distribution and Vitamin D, BMD, and Insulin Resistance in the NGT Group

#### 4.1.1. Correlation Between Adipose Distribution and Vitamin D in NGT Subjects

Adipose tissue serves as the primary reservoir for fat-soluble vitamin Vitamin D. Beyond simple storage, it expresses VDR and metabolic enzymes, positioning it as a direct target organ involved in the synthesis and catabolism of vitamin Vitamin D. In the NGT group, indicators of adiposity—including BMI, FAT%, and both VAT and SAT areas—exhibited significant negative correlations with serum 1,25(OH)_2_D_3_ levels. Notably, multivariate regression analysis identified VAT area as the most significant independent determinant of 1,25(OH)_2_D_3_ levels, suggesting that visceral adiposity plays a pivotal role in the metabolism of active vitamin D_3_.

Cumulative evidence indicates that while vitamin D concentrations are higher within the adipose tissue of obese individuals, their serum levels are paradoxically low. This phenomenon is likely attributed to the sequestration of vitamin D within fat stores, which reduces its systemic bioavailability [13]. Furthermore, some studies suggest a diminished release rate of 1,25(OH)_2_D_3_ from adipose tissue into the circulation in obese populations [14]. 1,25(OH)_2_D_3_ exerts its biological effects by binding to the VDR. Khan et al. demonstrated that VDR single-nucleotide polymorphisms correlate specifically with VAT rather than waist circumference or BMI, hinting that visceral fat may influence 1,25(OH)_2_D_3_ concentrations by modulating VDR expression [15]. Emerging data also suggest a regulatory feedback loop: 1,25(OH)_2_D_3_ modulates VDR expression in visceral pre-adipocytes in obese individuals, an effect not observed in subcutaneous fat or lean individuals [16]. Recent in vitro studies on mature human adipocytes showed that vitamin D3 is selectively retained, while its hydroxylated forms—25(OH)D and 1,25(OH)_2_D_3_—are rapidly cleared [17], further supporting the observed negative correlation between VAT and serum 1,25(OH)_2_D_3_.

During the progression of obesity, adipose expansion is characterized by low-grade inflammation and macrophage infiltration. Vitamin D appears to mitigate this by suppressing pro-inflammatory cytokines such as IL-6, IL-1β, IL-8, and MCP-1. Through VDR mediation, 1,25(OH)_2_D_3_ also induces adiponectin, exerting anti-inflammatory and insulin-sensitizing effects while reducing monocyte recruitment to adipose tissue [18]. Additionally, 1,25(OH)_2_D_3_ has been shown to reduce de novo lipogenesis and adipocyte size while promoting lipolysis in 3T3-L1 cells [19]. It also upregulates genes involved in fatty acid oxidation (PPARα, PGC1α, and CPT1α) [20]. By enhancing lipid catabolism and reducing synthesis, vitamin D effectively remodels adipose tissue, alleviating the metabolic stress and dysfunction associated with hypertrophic adipocytes [21].

#### 4.1.2. Correlation Between Adipose Distribution and BMD

Osteoporosis remains a significant public health burden, with obesity identified as a major modulator of BMD. However, the nexus between vitamin D, adipose tissue, and BMD remains contentious. While some data suggest that VAT adversely affects skeletal health, particularly in men [22], cross-sectional data from the Amirkola Health and Aging Project (AHAP) found that VAT remained positively correlated with BMD in the elderly after adjusting for age, sex, and BMI [23]. Conversely, larger studies in younger adults have identified a non-linear, U-shaped relationship between VAT and lumbar spine BMD [24].

Our study found no significant correlation between 1,25(OH)_2_D_3_ or 25(OH)D and BMD, which diverges from previous reports. For instance, Yang et al. reported that 1,25(OH)_2_D_3_ deficiency impairs osteoblastogenesis and accelerates bone loss, a process reversible via supplementation [25]. Other studies have linked 25(OH)D deficiency to increased fracture risk [26], though these associations may be sex-specific [27].

#### 4.1.3. Correlation Between Adipose Distribution and Insulin Resistance (IR)

Insulin resistance is the pathophysiological hallmark of diabetes, driven by the excessive release of free fatty acids (FFAs), reactive oxygen species (ROS), and pro-inflammatory cytokines from adipose tissue. This lipotoxicity disrupts mitochondrial and endoplasmic reticulum function, creating a deleterious cycle of systemic inflammation [28]. Our results indicate that in the NGT group, both VAT and SAT areas were significantly positively correlated with HOMA-IR and negatively correlated with the ISI, aligning with global epidemiological trends.

Interestingly, earlier research highlights that while both VAT and SAT contribute to IR, VAT acts as a more potent driver in women [29]. Studies using ultrasonography have further established VAT as an independent predictor of pre-diabetes, noting that for every unit increase in VAT, insulin sensitivity decreases by approximately 5%. Consistent with our findings, VAT appears to impair insulin sensitivity directly rather than affecting β-cell secretory function (HOMA-β) [30]. These results underscore the necessity for early intervention in euglycemic obese individuals to mitigate future diabetic risk.

### 4.2. Relationship Between Adipose Distribution and Vitamin D, BMD, and IR in the T2DM Group

#### 4.2.1. Correlation Between Adipose Distribution and Vitamin D

In the T2DM cohort, increased body weight and VAT area were associated with significantly lower levels of 25(OH)D and 1,25(OH)_2_D_3_. Multivariate analysis confirmed VAT area as the primary determinant of 1,25(OH)_2_D_3_ levels. Mechanistically, 1,25(OH)_2_D_3_ may regulate adipocyte apoptosis by triggering calcium-dependent signaling pathways [31]. Our findings align with studies suggesting that high vitamin D levels are protective against abdominal obesity [32]. Furthermore, vitamin D supplementation has been shown to downregulate lipogenic proteins (Acc, Fasn) and enhance fatty acid oxidation genes (CPT1A, PGC1α, and PPARα) [5,33,34]. Interestingly, animal models suggest that vitamin D’s capacity to reduce hepatic lipid accumulation may be more effective in lean phenotypes compared to obese ones [35]. Additionally, the duration of diabetes was not available for all participants. Since long-standing diabetes is associated with progressive renal tubular dysfunction and altered mineral metabolism (e.g., via the FGF23 pathway), this could potentially affect circulating 1,25(OH)_2_D_3_ levels. However, we have adjusted for eGFR and HbA1c in our final model, which serves as a proxy for disease severity and renal metabolic capacity [36].

#### 4.2.2. Correlation Between Adipose Distribution and BMD

Our analysis revealed that subjects with an excessive VAT area exhibited higher BMD and T-scores. While 25(OH)D was positively correlated with Z-scores, 1,25(OH)_2_D_3_ showed an unexpected negative correlation with BMD and T-scores. The literature often reports increased BMD in T2DM patients, possibly due to the anabolic effects of hyperinsulinemia. Because of structural homology, insulin can cross-react with IGF-1 receptors on osteoblasts, promoting bone formation [37]. However, the relationship between T2DM and BMD remains a subject of debate, with studies reporting varied outcomes (increased, decreased, or stable BMD) [38]. The finding that higher VAT corresponds with higher BMD suggests that visceral fat may influence bone through pathways independent of vitamin D. However, the ethnic homogeneity of our sample (Han Chinese from Yunnan) limits the generalizability of these findings.

#### 4.2.3. Correlation Between Adipose Distribution and IR

The T2DM group demonstrated significantly impaired β-cell function compared to the NGT and Control groups. VAT area was strongly associated with elevated HOMA-IR and reduced ISI, whereas SAT showed no significant correlation. These findings suggest that visceral adiposity, rather than subcutaneous fat, is the primary driver of insulin resistance in T2DM. Hypertrophic visceral adipocytes secrete an array of adverse adipokines (Leptin, IL-6, TNF-α, Vaspin) while downregulating adiponectin, thereby exacerbating glucose dysregulation and β-cell exhaustion [39,40].

### 4.3. Strengths, Limitations, and Outlook

This study provides critical insights into the role of visceral fat in 1,25(OH)_2_D_3_ metabolism; however, several limitations must be acknowledged. First, the study was confined to the Han Chinese population in Yunnan, which may limit the applicability of the results to other ethnic groups or regions. The NGT sample size (*n* = 61) was relatively small, warranting validation in larger cohorts. Second, the lack of data on menopausal status may confound the analysis of fat distribution and BMD. Third, 1,25(OH)_2_D_3_ has a significantly shorter circulating half-life (approximately 4–6 h) compared to 25(OH)D. Therefore, a single serum measurement may be susceptible to acute physiological fluctuations, which could potentially skew the results and may not reflect long-term vitamin D endocrine status.

In addition to metabolic factors, the role of mechanical loading in obesity cannot be overlooked. According to Wolff’s Law, increased body weight exerts greater mechanical stress on weight-bearing bones, which typically stimulates bone formation and increases BMD. While our study observed associations between 1,25(OH)_2_D_3_ and BMD, these effects should be interpreted alongside the protective physical impact of mechanical loading in individuals with higher BMIs. Furthermore, due to the retrospective nature of our clinical cohort, data for key regulatory factors such as PTH, serum calcium, and comprehensive renal function markers were missing for a significant portion of participants. To maintain adequate statistical power and avoid the bias associated with a substantially reduced sample size, these variables were excluded from the final multivariable regression models. Consequently, this research should be interpreted as an exploratory analysis aimed at identifying clinical trends rather than establishing definitive causal relationships. Future longitudinal studies should focus on prospective designs with more comprehensive data collection—including the PTH–calcium–vitamin D axis—and examine the specific molecular mechanisms by which different adipose depots interact with vitamin D and bone metabolism.

## 5. Conclusions

In both the NGT and T2DM groups, adiposity indices—including BMI, FAT%, VAT, and SAT—were inversely correlated with 1,25(OH)_2_D_3_ levels. However, multivariate regression analysis identified VAT as the primary determinant of 1,25(OH)_2_D_3_, suggesting that visceral adiposity plays a pivotal role in the metabolism of active vitamin D3.

In the context of visceral obesity, 1,25(OH)_2_D_3_ levels may be more closely associated with adiposity-related metabolic alterations than 25(OH)D.

The observed positive association between VAT and BMD in T2DM, despite lower 1,25(OH)_2_D_3_ levels, suggests that visceral fat may influence bone metabolism through more complicated mechanisms.

VAT exerts a disproportionately greater influence on insulin resistance than SAT in the T2DM population, highlighting its role as a key pathogenic factor for T2DM.

## Figures and Tables

**Table 1:** Baseline clinical and anthropometric characteristics of participants in the NGT and T2DM groups ($\bar{x}\pm s$).

Parameters	NGT Group (*n* = 61)	T2DM Group (*n* = 106)	*p*-Value
Age (years)	42.80 ± 11.77	51.95 ± 9.88	<0.001 **
Sex	21/40	61/45	0.014 *
Height (m)	1.61 ± 0.68068	1.65 ± 0.082	0.016 *
Weight (kg)	64.33 ± 13.15	67.62 ± 12.07	0.259
BMI (kg/m^2^)	24.68 ± 4.40	24.81 ± 3.62	0.966
WC (cm)	90.05 ± 12.72	92.33 ± 11.35	0.612
HC (cm)	97.53 ± 8.48	98.34 ± 10.26	0.913
WHR	0.92 ± 0.08	0.94 ± 0.07	0.329
Fat (%)	29.74 ± 10.01	30.73 ± 8.51	0.824
FMI (kg/m^2^)	7.53 ± 3.84	7.52 ± 3.39	0.793
VAT Area (cm^2^)	84.47 ± 43.49	95.26 ± 37.48	0.156
SAT Area (cm^2^)	196.12 ± 79.48	189.48 ± 75.23	0.832
VAT Area/SAT Area	0.45 ± 0.19	0.53 ± 0.17	0.011 *

*: *p* < 0.05, **: *p* < 0.01.

**Table 2:** Spearman correlation coefficients (r) between serum vitamin D metabolites and clinical/metabolic parameters in the NGT and T2DM groups.

Parameters	NGT		T2DM	
	25(OH)D	1,25(OH)_2_D_3_	25(OH)D	1,25(OH)_2_D_3_
Age (years)	0.057	0.039	−0.074	−0.028
Height (m)	−0.026	0.032	−0.184	−0.026
Weight (kg)	−0.109	−0.327 *	−0.233 *	−0.296 **
BMI (kg/m^2^)	−0.154	−0.373 **	−0.193	−0.336 **
WC (cm)	0.019	−0.235	−0.201	−0.405 **
HC (cm)	0.120	−0.239	−0.133	−0.267 **
WHR	0.015	−0.096	−0.167	−0.329 **
Fat (%)	0.000	−0.327 *	−0.112	−0.298 **
FMI (kg/m^2^)	−0.042	−0.402 **	−0.128	−0.342 **
VAT Area (cm^2^)	−0.138	−0.309 *	−0.202 *	−0.314 **
SAT Area (cm^2^)	−0.070	−0.381 **	−0.181	−0.331 **
VAT Area/SAT Area	−0.028	0.049	−0.042	−0.021
Ca (mmol/L)	0.119	0.108	0.055	−0.182
HbA1C (%)	−0.276	0.175	−0.161	0.045
HOMA-IR	0.061	−0.384	−0.168	−0.021
HOMA-β	0.118	0.158	0.011	0.106
ISI	−0.061	0.384	0.144	0.037
BMD1 (g/cm^2^)	−0.221	0.051	0.032	−0.285 *
BMD2 (g/cm^2^)	−0.271	−0.046	0.109	−0.237 *
BMD3 (g/cm^2^)	−0.272	−0.035	0.189	−0.228 *
BMD4 (g/cm^2^)	−0.268	0.077	0.100	−0.118
BMD (L1–L4) (g/cm^2^)	−0.267	0.008	0.074	−0.215
BMD (L2–L4) (g/cm^2^)	−0.261	0.002	0.134	−0.224 *
T1	−0.206	0.079	0.043	−0.244 *
T2	−0.178	0.078	0.108	−0.265 *
T3	−0.293 *	−0.017	0.182	−0.216
T4	−0.259	0.115	0.081	−0.109
T (L1–L4)	−0.262	0.058	0.158	−0.181
T (L2–L4)	−0.267	0.059	0.129	−0.169
Z1	−0.127	0.213	0.195	0.096
Z2	−0.045	0.203	0.198	0.011
Z3	−0.119	0.130	0.312 **	0.077
Z4	−0.167	0.242	0.217 *	0.180
Z (L1–L4)	−0.127	0.208	0.286 *	0.114
Z (L2–L4)	−0.129	0.189	0.287 *	0.118
PTH (pg/mL)	−0.272 *	0.094	−0.142	0.145

*: *p* < 0.05, **: *p* < 0.01.

**Table 3:** Spearman correlation coefficients (r) between VAT/SAT areas and relevant clinical/metabolic parameters in the T2DM group.

Parameters	VAT Area (cm^2^)	SAT Area (cm^2^)
Age (years)	−0.048	−0.018
Height (m)	0.302 **	0.089
Weight (kg)	0.731 **	0.665 **
BMI (kg/m^2^)	0.725 **	0.807 **
WC (cm)	0.718 **	0.777 **
HC (cm)	0.579 **	0.667 **
WHR	0.504 **	0.474 **
Fat (%)	0.583 **	0.714 **
FMI (kg/m^2^)	0.619 **	0.745 **
VAT Area/SAT Area	0.452 **	−0.317 **
HbA1C (%)	0.131	0.028
HOMA-IR	0.285 **	0.148
HOMA-β	−0.030	−0.004
ISI	−0.303 **	−0.142

**: *p* < 0.01.

**Table 4:** Comparison of clinical characteristics between participants with normal and excessive VAT areas in the NGT and T2DM groups ($\bar{x}\pm s$).

Parameters	NGT		*p*-Value	T2DM		*p*-Value
	Normal VAT Area	Excess VAT Area		Normal VAT Area	Excess VAT Area	
Age (years)	43.07 ± 11.58	42.06 ± 12.62	0.905	52.27 ± 9.56	51.54 ± 10.39	0.710
Height (m)	1.61 ± 0.06	1.61 ± 0.07	0.905	1.63 ± 0.08	1.67 ± 0.08	0.032 *
Weight (kg)	59.85 ± 10.58	76.38 ± 11.94	<0.001 **	61.15 ± 9.68	76.07 ± 9.38	<0.001 **
BMI (kg/m^2^)	22.98 ± 3.10	29.27 ± 4.12	<0.001 **	22.86 ± 2.77	27.35 ± 2.97	<0.001 **
WC (cm)	83.50 ± 9.06	102.54 ± 8.72	<0.001 **	85.75 ± 8.21	100.10 ± 9.50	<0.001 **
HC (cm)	94.62 ± 7.50	103.10 ± 7.65	<0.002 **	93.88 ± 6.30	103.61 ± 11.54	<0.001 **
WHR	0.88 ± 0.05	1.00 ± 0.08	<0.001 **	0.91 ± 0.06	0.97 ± 0.07	<0.001 **
Fat (%)	26.36 ± 9.13	37.96 ± 6.94	<0.001 **	26.27 ± 7.15	35.81 ± 6.99	<0.001 **
FMI (kg/m^2^)	6.22 ± 2.83	11.25 ± 3.24	<0.001 **	5.87 ± 2.37	9.40 ± 3.42	<0.001 **
VAT Area (cm^2^)	63.09 ± 19.73	141.94 ± 37.27	0.004 **	68.83 ± 19.89	129.74 ± 27.75	<0.001 **
SAT Area (cm^2^)	171.35 ± 63.36	262.69 ± 81.84	<0.001 **	152.60 ± 46.14	237.59 ± 78.97	<0.001 **
VAT Area/ SAT Area	0.40 ± 0.14	0.59 ± 0.23	0.004 **	0.48 ± 0.15	0.59 ± 0.17	0.001 **

*: *p* < 0.05, **: *p* < 0.01.

**Table 5:** Clinical Characteristics of Participants in the NGT and T2DM Groups, Stratified by BMI Category ($\bar{x}\pm s$).

Parameters	NGT		*p*-Value	T2DM		*p*-Value
	Normal BMI	Excess BMI		Normal BMI	Excess BMI	
Age (years)	43.96 ± 11.50	41.88 ± 12.07	0.562	51.70 ± 9.11	52.15 ± 10.53	0.817
Height (m)	1.59 ± 0.54	1.63 ± 0.08	0.064	1.64 ± 0.80	1.66 ± 0.83	0.221
Weight (kg)	53.56 ± 5.75	72.82 ± 10.90	<0.001 **	58.56 ± 8.12	74.84 ± 9.58	<0.001 **
BMI (kg/m^2^)	21.07 ± 1.79	27.53 ± 3.68	<0.001 **	21.79 ± 8.10	27.22 ± 2.63	<0.001 **
WC (cm)	77.45 ± 6.46	96.64 ± 9.86	<0.001 **	83.96 ± 7.43	98.56 ± 9.63	<0.001 **
HC (cm)	90.00 ± 3.79	101.48 ± 7.53	<0.001 **	92.78 ± 5.76	102.49 ± 10.94	<0.001 **
WHR	0.85 ± 0.52	0.95 ± 0.08	<0.001 **	0.90 ± 0.05	0.96 ± 0.07	<0.001 **
Fat (%)	23.01 ± 7.71	35.43 ± 8.07	<0.001 **	26.15 ± 6.19	33.99 ± 8.48	<0.001 **
FMI (kg/m^2^)	4.94 ± 1.99	10.01 ± 3.24	<0.001 **	5.63 ± 1.83	8.82 ± 3.61	<0.001 **
VAT Area (cm^2^)	59.46 ± 22.41	115.88 ± 32.51	<0.001 **	69.38 ± 25.38	115.88 ± 32.51	<0.001 **
SAT Area (cm^2^)	138.12 ± 49.95	228.78 ± 75.06	<0.001 **	140.15 ± 36.72	228.78 ± 75.06	<0.001 **
VAT Area/ SAT Area	0.46 ± 0.19	0.44 ± 0.19	0.609	0.52 ± 0.18	0.53 ± 0.15	0.604

**: *p* < 0.01.

**Table 6:** Comparison of vitamin D levels, bone metabolism indices, and clinical parameters between normal and excessive VAT subgroups in the NGT and T2DM groups [Median (P25, P75)].

Parameters	NGT		*p*-Value	T2DM		*p*-Value
	Normal VAT Area	Excess VAT Area		Normal VAT Area	Excess VAT Area	
Age (years)	43.96 ± 11.50	41.88 ± 12.07	0.562	51.70 ± 9.11	52.15 ± 10.53	0.817
Ca (mmol/L)	2.27 (2.20, 2.33)	2.22 (2.18, 2.32)	0.494	2.26 (2.15, 2.32)	2.33 (2.23, 2.40)	0.006 **
HbA1C (%)	5.10 (4.55, 5.28)	5.10 (4.70, 5.20)	0.921	7.50 (6.40, 10.10)	8.20 (6.80, 10.50)	0.477
HOMA-IR	1.80 (1.33, 2.52)	2.66 (1.70, 4.47)	0.059	1.80 (1.33, 2.52)	2.12 (1.64, 3.76)	0.020 *
HOMA-β	229.00 (−716.65, 353.46)	252.78 (102.71, 321.43)	0.963	51.79 (25.81, 101.27)	55.22 (31.08, 106.79)	0.784
ISI	0.71 (0.49, 0.79)	0.38 (0.23, 0.60)	0.059	0.56 (0.40, 0.75)	0.47 (0.27, 0.59)	0.011 *
BMD1 (g/cm^2^)	0.97 (0.91, 1.05)	1.04 (0.88, 1.10)	0.545	0.95 (0.89, 1.01)	1.02 (0.90, 1.08)	0.048 *
BMD2 (g/cm^2^)	1.07 (0.95, 1.14)	1.09 (1.04, 1.19)	0.275	1.04 (0.93, 1.11)	1.07 (0.98, 1.24)	0.030 *
BMD3 (g/cm^2^)	1.11 (1.02, 1.23)	1.12 (1.05, 1.20)	0.829	1.11 (1.04, 1.16)	1.13 (1.07, 1.24)	0.211
BMD4 (g/cm^2^)	1.10 (0.98, 1.19)	1.14 (0.97, 1.25)	0.437	1.08 (1.01, 1.17)	1.11 (0.98, 1.22)	0.447
BMD (L1–L4) (g/cm^2^)	1.05 (0.98, 1.16)	1.11 (1.00, 1.25)	0.469	1.05 (0.98, 1.23)	1.09 (1.02, 1.21)	0.053
BMD (L2–L4) (g/cm^2^)	1.08 (1.00, 1.18)	1.14 (1.05, 1.19)	0.326	1.08 (1.00, 1.16)	1.11 (1.03, 1.23)	0.170
T1	−0.80 (−1.27, −0.20)	−0.30 (−1.95, 0.23)	0.619	−1.20 (−1.90, −0.65)	−0.70 (−1.50, −1.20)	0.120
T2	−0.75 (−1.48, 0.08)	−0.35 (−1.25, 0.20)	0.665	−1.20 (−1.75, −0.70)	−0.70 (−1.50, 0.40)	0.035 *
T3	−0.45 (−1.15, 0.6)	−0.45 (−1.05, 0.53)	0.948	−0.70 (−1.15, −0.10)	−0.40 (−1.00, 0.45)	0.352
T4	−0.55 (−1.45, 0.38)	−0.05 (−1.85, 0.65)	0.713	−0.70 (−1.40, 0.05)	−0.50 (−1.40, 0.17)	0.380
T (L1–L4)	−0.65 (−1.35, 0.18)	−0.40 (−1.55, 0.38)	0.665	−1.00 (−1.35, −0.35)	−0.70 (−1.35, 0.08)	0.270
T (L2–L4)	−0.50 (−1.38, 0.28)	−0.20 (−1.35, 0.33)	0.642	−0.90 (−1.30, −0.20)	−0.60 (−1.25, 0.18)	0.337
Z1	−0.60 (−1.78, −0.03)	−2.10 (−2.83, 0.2)	0.166	−0.60 (−1.35, 0.05)	−1.15 (−1.90, −0.53)	0.042 *
Z2	−0.60 (−1.70, 0.28)	−1.55 (−2.55, −0.58)	0.076	−0.80 (−1.55, −0.20)	−1.05 (−1.88, −0.15)	0.417
Z3	−0.25 (−1.28, 0.65)	−1.35 (−2.52, −0.33)	0.035 *	−0.20 (−1.00, 0.50)	−0.70 (−1.48, −0.03)	0.070
Z4	−0.40 (−1.43, 0.10)	−1.80 (−2.95, 0.43)	0.103	−0.30 (−1.10, 0.60)	−1.00 (−1.58, −0.13)	0.054
Z (L1–L4)	−0.65 (−1.57, 0.15)	−1.85 (−2.68, −0.13)	0.092	−0.40 (−0.25, 0.10)	−1.00 (−1.75, −0.20)	0.081
Z (L2–L4)	−0.55 (−1.40, 0.28)	−1.60 (−2.53, −0.13)	0.065	−0.40 (−1.15, 0.20)	−0.85 (−1.65, −0.15)	0.091
PTH (pg/mL)	50.54 (24.09, 60.35)	51.67 (7.83, 41.06)	0.645	40.63 (28.54, 58.44)	44.06 (22.47, 61.35)	0.795
25(OH)D (ng/mL)	16.99 (14.56, 21.12)	16.96 (15.09,21.82)	0.973	16.47 (13.32, 21.14)	14.34 (12.74, 17.86)	0.640
1,25(OH)_2_D_3_ (fmol/mL)	660.31 (408.35, 942.10)	409.77 (334.95, 668.31)	0.049 *	631.83 (533.23, 954.21)	500.58 (368.45, 710.10)	0.006 **

*: *p* < 0.05, **: *p* < 0.01.

**Table 7:** Comparison of vitamin D metabolites, bone metabolism markers, and clinical cha-racteristics between normal BMI and excess BMI subgroups in the NGT and T2DM groups [Median (P25, P75)].

Parameters	NGT		*p*-Value	T2DM		*p*-Value
	Normal BMI	Excess BMI		Normal BMI	Excess BMI	
Ca (mmol/L)	2.27 (2.19, 2.32)	2.24 (2.19, 2.34)	0.974	2.28 (2.19, 2.34)	2.29 (2.19, 2.36)	0.771
HbA1C (%)	5.00 (4.48, 5.23)	5.20 (4.70, 5.20)	0.527	7.85 (6.55, 10.28)	8.00 (6.43, 10.10)	0.706
HOMA-IR	1.25 (1.16, 1.41)	2.10 (1.47, 3.79)	0.047 *	1.77 (1.30, 2.38)	2.12 (1.54, 3.25)	0.023 *
HOMA-β	229.00 (−1540.00, 405.50)	187.33 (17.20, 306.43)	1.000	44.44 (21.32, 97.38)	61.39 (35.25, 118.00)	0.173
ISI	0.80 (0.71, 0.86)	0.48 (0.26, 0.68)	0.047 *	0.56 (0.41, 0.76)	0.47 (0.31, 0.65)	0.035 *
BMD1 (g/cm^2^)	0.96 (0.90, 1.03)	1.03 (0.88, 1.14)	0.108	0.93 (0.84, 1.00)	1.01 (0.92, 1.08)	0.024 *
BMD2 (g/cm^2^)	1.00 (0.96, 1.11)	1.13 (0.99, 1.19)	0.036 *	1.01 (0.93, 1.08)	1.11 (0.99, 1.20)	0.005 **
BMD3 (g/cm^2^)	1.10 (1.03, 1.21)	1.15 (1.02, 1.24)	0.231	1.10 (1.03, 1.15)	1.13 (1.06, 1.24)	0.110
BMD4 (g/cm^2^)	1.07 (0.97, 1.19)	1.11 (1.00, 1.19)	0.227	1.07 (1.01, 1.17)	1.13 (1.00, 1.23)	0.120
BMD (L1–L4) (g/cm^2^)	1.03 (0.98, 1.13)	1.13 (1.00, 1.17)	0.119	1.04 (0.95, 1.10)	1.09 (1.01, 1.18)	0.037 *
BMD (L2–L4) (g/cm^2^)	1.07 (0.99, 1.16)	1.16 (1.03, 1.20)	0.085	1.07 (0.98, 1.11)	1.12 (1.03, 1.22)	0.027 *
T1	−0.90 (−1.10, −0.40)	−0.40 (−1.90, 0.60)	0.242	−1.25 (−1.98, −0.55)	−0.90 (−1.45, −0.20)	0.075
T2	−1.00 (−1.50, −0.30)	−0.20 (−1.10, 0.50)	0.034 *	−1.40 (−1.95, −0.83)	−0.70 (−1.55, 0.20)	0.014 *
T3	−0.60 (−0.90, 0.30)	0.10 (−1.30, 0.70)	0.447	−0.65 (−1.30, −0.30)	−0.40 (−1.00, 0.40)	0.220
T4	−0.60 (−1.50, 0.40)	−0.10 (−1.70, 0.50)	0.540	−0.80 (−1.25, 0.05)	−0.40 (−1.50, 0.30)	0.326
T (L1–L4)	−0.70 (−1.20, −1.00)	−0.20 (−1.60, 0.40)	0.330	−1.00 (−1.70, −0.53)	−0.70 (−1.25, 0.05)	0.131
T (L2–L4)	−0.70 (−1.20, 0.10)	−0.10 (−1.50, 0.40)	0.320	−0.95 (−1.53, −0.40)	−0.50 (−1.20, 0.25)	0.109
Z1	−0.50 (−1.40, −0.30)	−1.70 (−2.80, 0.20)	0.227	−0.70 (−1.30, −0.15)	−1.10 (−1.90, −0.45)	0.073
Z2	−0.70 (−1.70, −0.10)	−1.30 (−2.50, 0.20)	0.425	−0.80 (−1.58, −0.23)	−1.00 (−1.85, −0.10)	0.687
Z3	−0.20 (−0.80, 0.50)	−1.20 (−1.90, 0.20)	0.090	−0.20 (−0.95, 0.40)	−0.70 (−1.45, 0.15)	0.084
Z4	−0.20 (−1.10, 0.20)	−1.20 (−2.10, 0.10)	0.064	−0.25 (−1.08, 0.28)	−1.00 (−1.55, 0.45)	0.066
Z (L1–L4)	−0.50 (−1.20, 0.00)	−1.60 (−2.40, 0.20)	0.122	−0.45 (−1.10, 0.00)	−1.00 (−1.70, −0.20)	0.101
Z (L2–L4)	−0.40 (−1.00, 0.20)	−1.40 (−2.30, 0.30)	0.088	−0.40 (−1.00, 0.18)	−1.00 (−1.15, 0.05)	0.107
PTH (pg/mL)	50.83 (23.90, 59.20)	51.74 (36.54, 59.44)	0.760	39.78 (28.52, 57.78)	43.88 (25.09, 61.25)	0.792
25(OH)D (ng/mL)	18.33 (16.40, 21.18)	16.02 (12.80, 20.71)	0.060	16.78 (13.18, 21.22)	15.04 (12.86, 18.76)	0.127
1,25(OH)_2_D_3_ (fmol/mL)	748.24 (456.92, 988.16)	411.88 (357.21, 731.36)	0.008 **	643.97 (533.03, 961.64)	517.23 (367.24, 746.22)	0.017 *

*: *p* < 0.05, **: *p* < 0.01.

**Table 8:** Multivariate Regression Analysis Identifying Predictors of 1,25(OH)_2_D_3_ in the NGT and T2DM groups.

Variable	NGT Coefficient (B)	*p*-Value	T2DM Coefficient (B)	*p*-Value
Constant	957.389	<0.001	1060.190	<0.001
VAT Area	−0.333	0.021	−0.398	0.001

## Data Availability

The raw data supporting the conclusions of this article will be made available by the authors on request.

## Supplementary Materials

The following supporting information can be downloaded at https://www.mdpi.com/article/10.3390/metabo16060379/s1, Figure S1 Correlation heatmap illustrating the associations between vitD and BMD in the male and female; Table S1 Information on the analyte.

## Abbreviations

The following abbreviations are used in this manuscript:

BMD	Bone Mineral Density
Ca	Calcium
DXA	Dual X-ray Absorptiometry
Fat%	Body Fat Percentage
FMI	Fat Mass Index
FPG	Fasting Plasma Glucose
HbA1C%	Glycated Hemoglobin
HC	Hip Circumference
HOMA-IR	Homeostasis Model Assessment of Insulin Resistance
HOMA-β	Pancreatic β-cell Function Index
ISI	Insulin Sensitivity Index
NGT	Normal Glucose Tolerance
PTH	Parathyroid Hormone
SAT	Subcutaneous Adipose Tissue Areas
T2DM	Type 2 Diabetes Mellitus
VAT	Visceral Adipose Tissue Areas
WC	Waist Circumference
WHR	Waist-to-Hip Ratio
25(OH)D	25-hydroxyvitamin D
1,25(OH)_2_D_3_	1,25-dihydroxyvitamin D3
2h-PG	2h Postprandial Glucose
