# Supplementary material for: Relationships Among Adipose Tissue Distribution, Vitamin D, and Bone Metabolism in Normoglycemic and Type 2 Diabetic Individuals
Source: Metabolites. 2026 May 31;16(6):379. doi: 10.3390/metabo16060379 (PMC13304412; doi:10.3390/metabo16060379)
Supplement: Supplementary file 1 [file metabolites-16-00379-s001.zip › metabolites-4300820-supplementary.pdf]

Supplementary Table S1 Etailed information on the analyte

Analyte	Kit Manufacturer	Detection Range	Sensitivity	Intra-assay CV	Inter-assay CV
1,25(OH)2D3	CUSABIO	1000 - 5000 fmol/L	250 fmol/L	⩽ 3.0%	3.3%
25(OH)D	Roche	3-120ng/mL	< 5 ng/mL	⩽ 3.0%	3.3%
PTH	Roche	15-65pg/mL	< 1.2 ng/mL	1.5-3.3%	2.3-4.4%
HbA1C (%)	Arkray Inc.	3.0 - 20.0 %	< 1.0 %	≤ 0.7%	≤ 0.6%
C-peptide	Siemens Healthcare	0.1 - 20 ng/mL	0.08 ng/mL	1.7% - 2.8%	3.5% - 6.3%
Insulin	Siemens Healthcare	0.5 - 300 mU/L	0.5mU/L	3.5% - 8.0%	4.5% - 8.7%

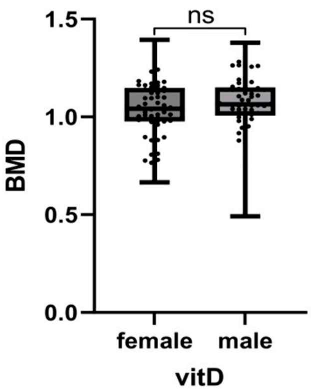

Supplementary Figure S1 Correlation heatmap illustrating the associations between vitD and BMD in the male and female groupsgroups.
